# Cell-Free (RNA) and Cell-Associated (DNA) HIV-1 and Postnatal Transmission through Breastfeeding

**DOI:** 10.1371/journal.pone.0051493

**Published:** 2012-12-28

**Authors:** James Ndirangu, Johannes Viljoen, Ruth M. Bland, Siva Danaviah, Claire Thorne, Philippe Van de Perre, Marie-Louise Newell

**Affiliations:** 1 Africa Centre for Health and Population Studies, University of KwaZulu-Natal, Somkhele, South Africa; 2 University of Glasgow School of Medicine, Glasgow, United Kingdom; 3 MRC Centre of Epidemiology for Child Health, University College London Institute of Child Health, London, United Kingdom; 4 INSERM U, Montpellier, France; 5 Université Montpellier, Montpellier, France; 6 Département de Bactériologie-Virologie et Département d’Information Médicale, CHU Montpellier, Montpellier, France; University of Cape Town, South Africa

## Abstract

**Introduction:**

Transmission through breastfeeding remains important for mother-to-child transmission (MTCT) in resource-limited settings. We quantify the relationship between cell-free (RNA) and cell-associated (DNA) shedding of HIV-1 virus in breastmilk and the risk of postnatal HIV-1 transmission in the first 6 months postpartum.

**Materials and Methods:**

Thirty-six HIV-positive mothers who transmitted HIV-1 by breastfeeding were matched to 36 non-transmitting HIV-1 infected mothers in a case-control study nested in a cohort of HIV-infected women. RNA and DNA were quantified in the same breastmilk sample taken at 6 weeks and 6 months. Cox regression analysis assessed the association between cell-free and cell-associated virus levels and risk of postnatal HIV-1 transmission.

**Results:**

There were higher median levels of cell-free than cell-associated HIV-1 virus (per ml) in breastmilk at 6 weeks and 6 months. Multivariably, adjusting for antenatal CD4 count and maternal plasma viral load, at 6 weeks, each 10-fold increase in cell-free or cell-associated levels (per ml) was significantly associated with HIV-1 transmission but stronger for cell-associated than cell-free levels [2.47 (95% CI 1.33–4.59) vs. aHR 1.52 (95% CI, 1.17–1.96), respectively]. At 6 months, cell-free and cell-associated levels (per ml) in breastmilk remained significantly associated with HIV-1 transmission but was stronger for cell-free than cell-associated levels [aHR 2.53 (95% CI 1.64–3.92) vs. 1.73 (95% CI 0.94–3.19), respectively].

**Conclusions:**

The findings suggest that cell-associated virus level (per ml) is more important for early postpartum HIV-1 transmission (at 6 weeks) than cell-free virus. As cell-associated virus levels have been consistently detected in breastmilk despite antiretroviral therapy, this highlights a potential challenge for resource-limited settings to achieve the UNAIDS goal for 2015 of eliminating vertical transmission. More studies would further knowledge on mechanisms of HIV-1 transmission and help develop more effective drugs during lactation.

## Introduction

Globally, in 2010, an estimated 2.7 million people became infected with human immunodeficiency virus (HIV); 1.9 million (70%) of new infections occurred in sub-Saharan Africa (SSA) [1]. An estimated 390,000 (340,000–450,000) new infections occurred in children, 90% of these in SSA, mainly through mother-to-child transmission (MTCT) [1]. MTCT can occur before, during and after delivery, with postnatal transmission through breastfeeding which accounts for one-third to one-half of MTCT remaining an unresolved issue [2]. With maternal antiretroviral therapy (ART), the risk of MTCT can be substantially reduced [3]–[5]. However, ART is not always available in resource-limited settings with high HIV prevalence, where breastfeeding is the norm for infant survival, and where the provision of ART to the mother or the infant for up to one year of breastfeeding as per the current WHO guidelines [6] poses a challenge. Therefore, postnatal transmission of HIV-1 through breastfeeding is likely to remain an issue for the foreseeable future in resource-limited settings.

Although factors associated with MTCT have been quantified [7]–[10], the mechanisms underlying postnatal transmission remain poorly understood, in particular the relative roles of cell-free (RNA) and cell-associated (DNA) HIV-1 in breastmilk transmission. High levels of cell-free virus in maternal plasma and breastmilk are associated with a high risk of HIV-1 transmission during breastfeeding [11]–[16]. Similarly, an association has been observed with cell-associated virus in breastmilk, suggesting both cell-free and cell-associated are involved in breastmilk HIV-1 transmission [11], [17], [18]. We previously showed that cumulative exposure to RNA particles in breastmilk significantly increased the risk of HIV-1 acquisition postnatally independently from maternal antenatal CD4 cell count, plasma HIV-1 load, child sex and duration of breastfeeding [19]. Recent studies observe that while ART leads to undetectable levels of cell-free HIV-1 virus, cell-associated virus levels are still detected in breastmilk [20], [21]. Additionally, there are suggestions that cell-free and cell-associated virus vary in their prediction of HIV-1 transmission at early and late lactation stages [18]. If studies confirm such variations in HIV-1 transmission, and cell-associated virus levels are barely affected by maternal ART, this could account for the residual HIV-1 transmission during lactation.

This study examines the prevalence of, and quantifies the relationship between, cell-free and cell-associated shedding of HIV-1 virus in breastmilk and the risk of postnatal HIV-1 transmission, in both right and left breasts over the first 6 months postpartum.

## Materials and Methods

### Study Population

HIV-infected and HIV-uninfected women were enrolled in an intervention cohort study, between August 2001 and September 2004 [22], [23], to investigate whether breastfeeding in a high HIV prevalence, poor rural setting in South Africa could be made safe in terms of both HIV-1 transmission and infant morbidity and mortality. Weekly home visits documented infant feeding and morbidity while clinic follow-up of the infants and mothers were scheduled monthly between 6 weeks and 9 months. Ten milliliters of breastmilk were collected from each breast for HIV-infected and uninfected breastfeeding mothers at each scheduled clinic visit. Samples were transported and maintained at 4 degrees Celsius overnight and stored long-term as whole breastmilk at minus 80 degrees Celsius until testing.

A dried blood spot for each infant was collected at each visit and stored at minus 20 degrees Celsius. HIV-1 RNA quantification was performed using the Nuclisens HIV-1 QT assay (Organon Teknika, Boxtel, Netherlands) and Nuclisens EasyQ HIV-1 assay (Biomerieux, Boxtel, Netherlands) with a sensitivity of 80 copies HIV-1 RNA per ml of blood (equivalent to 1600 copies HIV-1 RNA per 50 µl dried blood spot) [24]. Rates of MTCT of HIV-1 during breastfeeding have been described previously [23]. Children were considered infected through breastfeeding if they had a negative HIV polymerase chain reaction (PCR) assay at 6 weeks of age and a positive PCR at any time thereafter. Single-dose nevirapine (sdNVP) for use during labour/delivery was provided for all HIV-infected women and to their newborns; ART for treatment or as MTCT prophylaxis from early in pregnancy or during the postnatal period was not available in the public health setting at the time of this study. Maternal viral load and CD4 count were collected antenatally. The project was approved by the Biomedical Ethics Review Committee (BREC) at the University of KwaZulu-Natal South Africa.

### Study Design

A case-control study was nested in this intervention cohort [22]. The primary study identified 42 babies who had acquired HIV infection postnatally (as diagnosed by PCR conversion) [23]. Our study includes 36 postnatally infected children who had both cell-free and cell-associated data on samples at 6 weeks and 6 months, and who were matched to controls. Cases and controls were matched (in a 1∶1 ratio) on infant age at breastmilk sampling with a maximum allowance of 2 weeks of the sample date of the case to reduce potential bias of varying concentrations of breastmilk RNA and DNA over time [25]. Cases were mothers who transmitted HIV-1 to their infants through breastmilk between 6 and 28 weeks postpartum while controls were non-transmitting HIV-1 infected mothers. Transmission was estimated to have occurred at the midpoint between an infant’s last HIV negative PCR test and first positive result. Infants were included if they had at least one cell-free and one cell-associated breastmilk sample available close to the estimated time of transmission (ETT). Breastmilk samples from both breasts, for postnatal transmitters and controls had DNA quantified twice (at 6 weeks and 6 months) and RNA at multiple time points before 6 months. Thirty-six transmitting mothers had 85 samples tested for HIV-1 RNA and DNA in both left and right breast; 36 control mothers had 81 samples. This study differs from the previous study which investigated the association between postnatal HIV acquisition at 6–28 weeks and cumulative cell-free HIV exposure (i.e. the overall amount of cell-free viral particles ingested by the infant during breastfeeding, upto infection or equivalent age of control) [19]. The volume of milk ingested per day was estimated by pattern of feeding and the probability of transmission estimated per liter of breastmilk ingested. However, that study did not access the influence of cell-associated virus integrated in latent T cells on postnatal transmission. In contrast, the current study presents the association between cell-free and cell-associated shedding of HIV-1 virus in breastmilk and postnatal HIV-1 transmission.

### Quantification of HIV-1 Cell-free and Cell-associated Virus

Cell-free HIV-1 quantification on breastmilk samples was performed as described previously [19]. Cell-associated HIV-1 quantification on whole breastmilk samples was performed using the Generic HIV DNA Cell assay (Biocentric, Bandol, France). Breastmilk samples were thawed at room temperature and vortex mixed. A maximum of 1.5 ml (range 0.5–1.5 ml) of breastmilk was aliquoted into a 2 ml microtube, centrifuged at 2000 g for 15 min and the lactoserum-lipid layer was removed to a 1.5 ml microtube. The lactoserum-lipid fraction was stored at −80°C. The remaining breastmilk pellet was used in the HIV DNA real time PCR (qPCR) assay. RNA was isolated from 500 µL of lactoserum with use of the magnetic particle-based ASPS method (Abbott), and HIV load was quantified using the Generic HIV Charge Virale assay (Biocentric, Bandol, France) on the MJ MiniOpticon quantitative PCR detection platform (Biorad), with a sensitivity of 375 copies per mL of lactoserum [26]. This method enabled accurate assessment of cell-free viral load entrapped by lipids [27]. The Qiagen DNA Mini Kit was used to isolate total DNA from the dry breastmilk pellet according to the manufacturer’s instructions. Total DNA concentration was measured with the Nanodrop instrument using 1 µl of sample. Samples with a DNA concentration of <50****ng/µl were tested neat. For samples with a DNA concentration of >50 ng/µl an appropriate dilution of up to 1∶10 was performed. The total reaction volume was 50 µl with a 20 µl sample input volume, according to manufacturer’s instructions.

The human GAPDH housekeeping gene (Primer_F : 5′-AAGGTCGGAGTCAACGGATT-3′; Primer_R R: 5′-CTCCTGGAAGATGGTGATGG-3′) was quantified by real-time PCR using SybrGreen to verify the integrity of the extracted DNA, to determine the presence or absence of inhibitors/contaminants, and to act as a reference gene for quantitative analysis [28]–[30]. Quantifying the host gene GAPDH provided an estimate of the number of cells per PCR, allowing expression of the number of copies of HIV per 10^6^ cells in our sample despite not having a cell count.

### Statistical Analysis

The analyses included transmitters and controls with both cell-free and cell-associated results available from the same breastmilk sample at 6 weeks and 6 months. When the 6 months results were more than 4 weeks after transmission, the RNA result closest to the transmission was used (RNA was quantified at multiple time points) while the average between the two DNA results was calculated, otherwise the result at 6 months was used. Breastmilk HIV-1 RNA viral load levels below the lower detectable limit (375 copies/ml) were assigned a value at the midpoint between this and zero (187.5 copies/ml) [31], [32]. Breastmilk HIV-1 DNA samples below the lower detectable limit were normalized for the amount of cells used to isolate the DNA (based on the GAPDH measurement which is different for each cell) [33]. No breastmilk samples were excluded because of low cell counts as all samples had DNA values above zero. Cell-free and cell-associated virus levels were analyzed on a decimal logarithmic scale to base-10 [11], [18]. Counts of DNA quantified per million cells were converted to concentrations of DNA per milliliter by multiplying by 0.08×10^6^ at 6 weeks and 0.05×10^6^ at 6 months breastmilk cells per milliliter, as suggested in previous studies [11], [34].

Chi-square test assessed differences in categorical variables while Wilcoxon rank-sum test was used for non-parametric analysis of continuous variables. Spearman rank correlation estimated correlation between continuous variables. Cox regression models, pooling multiple measurements from the left and right breastmilk samples, assessed the association between breastmilk cell-free and cell-associated virus levels and risk of postnatal HIV-1 transmission. Observation time was taken from 6 weeks of age (last negative HIV PCR assay) to the estimated time of HIV-1 infection or end of observation (6 months of age), whichever came first. Multivariable models included maternal antenatal CD4 cell count and plasma RNA [18], and were stratified by time (6 weeks and 6 months) because there are more infected cells in early than mature breastmilk [11]. The model adjusting for both antenatal CD4 count and viral load represented the best fit of the data using BIC and was thus retained as the final model. Data were analysed using Stata Version 11.2 (2009 StataCorp, College Station, Texas, USA).

## Results

**Figure 1:**
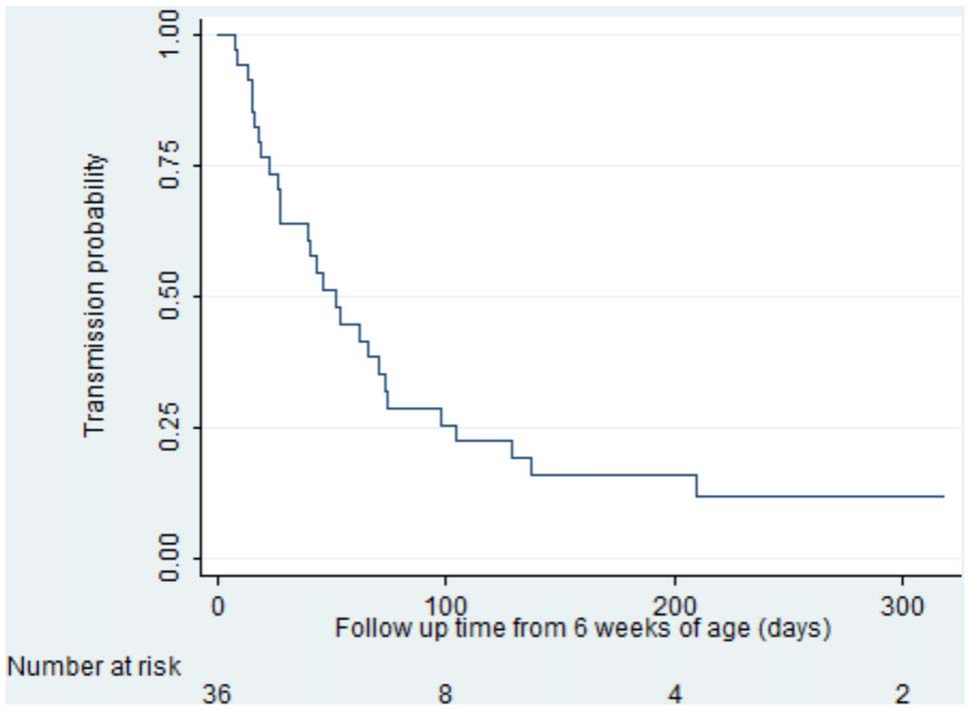
Kaplan-Meier curve showing transmission probabilities after 6 weeks of age.

A total of 166 HIV-1 RNA and DNA samples were included in this analysis from 72 mothers (36 in each of transmitters and controls); 81% predominantly breastfed (infants mainly received breastmilk plus water or water-based drinks but no other milk or food based fluid) for the first 6 months. 13.9% (5 of 36) of transmitting mothers had RNA and DNA below lower detectable limit in the last available breastmilk sample before transmission occurred. Transmitting mothers were more likely to have lower antenatal CD4 cell counts (p<0.001) and higher plasma viral load (p<0.001) than controls (Table 1). The median time to transmission in the cases was 85 (IQR 66–114) days (Figure 1).

**Table 1:** Baseline characteristics of Infants and HIV-positive mothers transmitting HIV-1 through breastmilk and their controls.

Infant or Maternal characteristic	Cases (n = 36)	Controls (n = 36)	*P*
**Age (years), median (IQR)**	25.5 (22.2–28.2)	27.3 (21.1–29.9)	0.502
**Antenatal CD4 count cells/µL, median (IQR)**	337 (198–540)	524 (369–697)	<0.001
**Antenatal viral load log_10_ copies/mL, median (IQR)**	4.5 (4.2–5.0)	4.0 (3.5–4.6)	<0.001
**Water source**			
Non-piped	13 (36.1)	11 (30.6)	0.714
Piped	23 (63.9)	25 (69.4)	
**Enrollment clinic**			
Rural	16 (44.4)	14 (38.9)	0.764
Peri-urban	11 (30.6)	12 (33.3)	
Urban	9 (25.0)	10 (27.8)	
**Maternal education**			
None	2 (5.6)	5 (13.9)	
Some primary	12 (33.3)	14 (38.9)	
Secondary and above	22 (61.1)	17 (47.2)	0.226
**Birth weight (grams), median (IQR)**	3200 (2800–3400)	3100 (2800–3500)	0.572
**Birth head circumference, median (IQR)**	34.8 (33.5–36.4)	35.0 (33.6–36.0)	0.828

Chi-square test assessed differences in categorical variables while Wilcoxon rank-sum test was used for non-parametric analysis of continuous variables.

Abbreviations: Cases, HIV-1 infected postnatal transmitters; Controls, non-transmitting HIV-1 infected mothers.

Across all samples tested, cell-free virus was above detectable limit in 76.5% (65/85) of breastmilk samples in the 36 transmitters and in 55.6% (44/81) in controls (p = 0.004); at 6 weeks and 6 months, prevalence was 79.1% and 73.8% in transmitters and 60.9% and 50.0% in controls, respectively. Overall, cell-associated virus was above detectable limit in 76.5% (65/85) of breastmilk samples in transmitters and in 45.7% (37/81) in controls (p<0.001); at 6 weeks and 6 months, prevalence was 76.7% and 76.5% in transmitters and 46.3% and 45.0% in controls, respectively. The detection levels of cell-free and cell-associated virus were similar in right and left breast; 43.8% and 56.9% (p = 0.092), respectively, for cell-free virus and 67.5% and 55.8% (p = 0.122), respectively, for cell-associated virus.

### HIV-1 RNA and DNA Loads in Breastmilk

**Figure 2:**
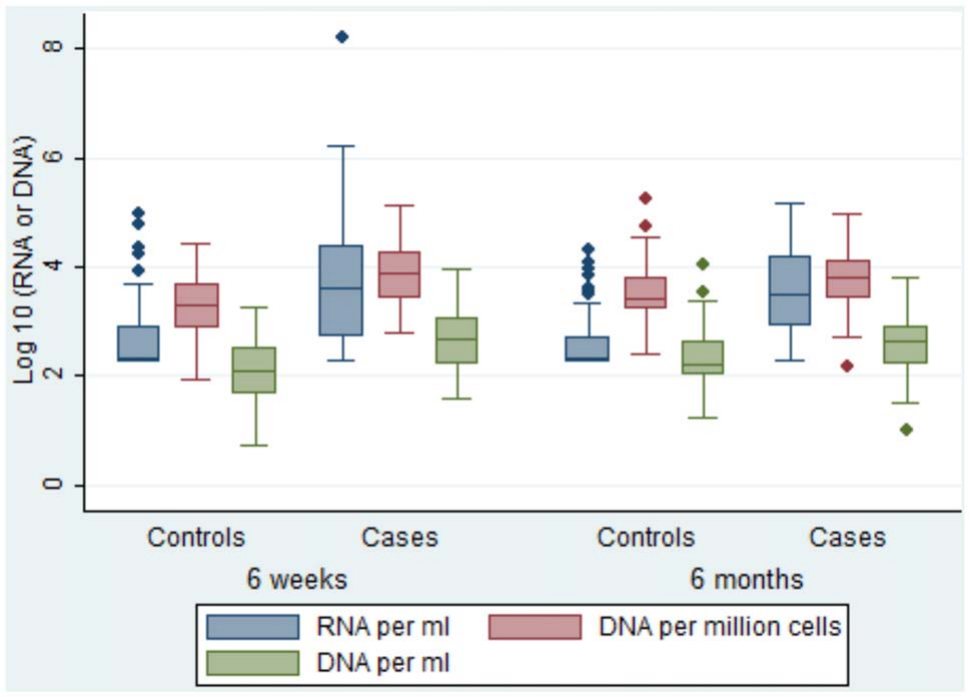
Distribution of observed log_10_ cell-free and cell-associated values by age of infant.

Cell-free virus levels ranged from below detection to a maximum of 1,590,000 copies per ml at 6 weeks and 6 months; cell-associated virus levels ranged from below detection to a maximum of 137,441 copies per ml. Median log_10_ cell-free values per milliliter were higher than cell-associated values per milliliter (2.8 vs. 2.3 at 6 weeks; p<0.001 and 2.7 vs. 2.4 at 6 months; p<0.001, respectively). Transmitting mothers had significantly higher log_10_ values of cell-free (median: 3.6 vs. 2.7; p<0.001 at 6 weeks and 3.5 vs. 2.3; p<0.001 at 6 months) and cell-associated per milliliter (median: 2.7 vs. 2.1; p<0.001 at 6 weeks and 2.6 vs. 2.2; p<0.001 at 6 months) values than controls (Figure 2).

**Figure 3:**
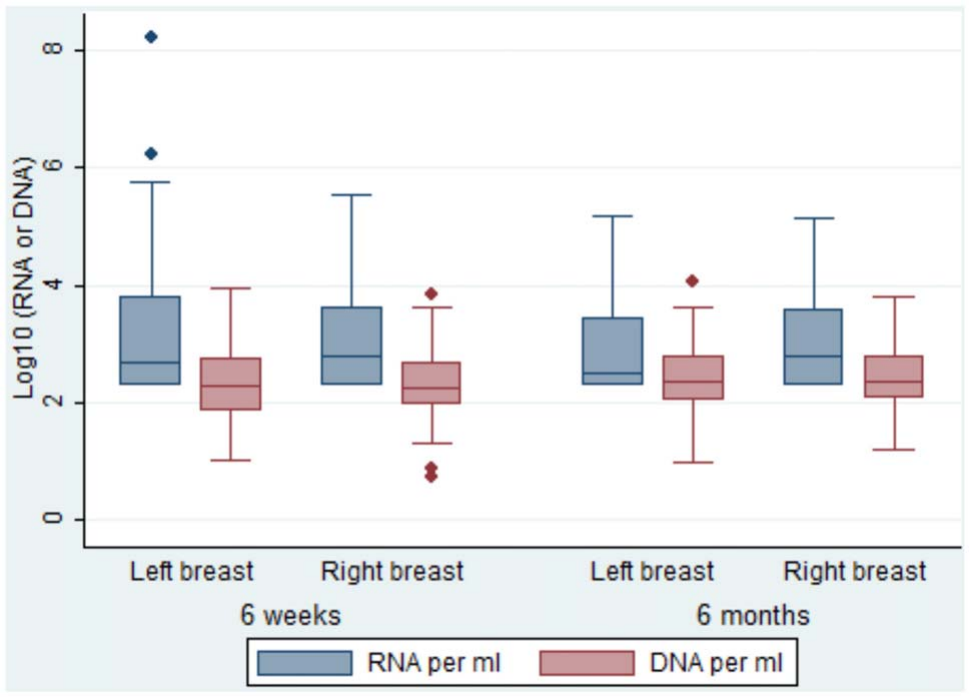
Distribution of log10 cell-free and cell-associated values (per ml) by breast and infant age.

The breastmilk cell-free and cell-associated levels were similar between breasts at both time points in the first 6 months postpartum (Figure 3). Overall, cell-free virus per milliliter and cell-associated virus levels per million cells were significantly positively correlated (*r* = 0.34, p<0.001); these correlation was maintained at 6 weeks (*r* = 0.37, p<0.001) and at 6 months (*r* = 0.32, p<0.001). Similarly, a positive correlation was obtained between cell-free virus per milliliter and cell-associated virus per milliliter (*r* = 0.33, p<0.001); these correlation was also maintained at 6 weeks (*r* = 0.38, p<0.001) and at 6 months (*r* = 0.32, p<0.001). Breastmilk cell-free virus levels were positively correlated with antenatal maternal plasma viral load (overall RNA: *r* = 0.46, p<0.001; at 6 weeks *r* = 0.46, p<0.001 and at 6 months *r* = 0.47, p<0.001) and negatively with maternal CD4 cell count (RNA: *r* = −0.44, p<0.001; at 6 weeks *r* = −0.43, p<0.001 and at 6 months *r* = −0.46, p<0.001). Similarly, breastmilk cell-associated virus levels per milliliter were positively correlated with antenatal maternal plasma viral load (overall DNA: *r* = 0.30, p<0.001; at 6 weeks *r* = 0.35, p<0.001 and at 6 months *r* = 0.26, p<0.001) and negatively with maternal CD4 cell count (DNA: *r* = −0.33, p<0.001; at 6 weeks *r* = −0.37, p<0.001 and at 6 months *r* = −0.29, p<0.001). Log_10_ cell-free and cell-associated virus levels in breastmilk were significantly higher in mothers with antenatal CD4 count below 500 compared to those with at least 500 cells per mm^3^ (median: 3.2 vs. 2.7, p<0.001 for cell-free and 3.7 vs. 3.3, p<0.001 for cell-associated virus levels).

### Correlation with HIV-1 Transmission

In univariate Cox analysis, each 10-fold increase in the average (between 6 weeks and 6 months) cell-free and cell-associated levels was associated with a significant 2- and a 4-fold increase in breastmilk transmission (HR 2.18 (95% confidence interval (CI) 1.66–2.87) and 4.18 (95% CI 2.24–7.79) respectively). Multivariably, adjusting for antenatal CD4 count and maternal plasma viral load, each 10-fold increase in cell-free or cell-associated levels was significantly associated with an approximate 2-fold increase in breastmilk transmission [adjusted hazard ratio (aHR) 1.96 (95% confidence interval (CI), 1.17–1.96) and 2.57 (95% CI 1.26–5.28) respectively].

A Cox regression model was fitted for the HIV-1 RNA and DNA breastmilk samples collected at both 6 weeks and 6 months to control for potential variation in levels over time [11]. The trend suggested that at 6 weeks, HIV-1 DNA levels in breastmilk were more important for HIV-1 transmission than RNA (aHR 1.98 vs. 1.16, both P>0.05). Conversely, at 6 months, RNA levels were more important than DNA (aHR 1.99 vs. 1.21, both p>0.05). However, statistical power was lost, possibly due to collinearity between the 6 weeks and 6 months HIV-1 RNA and DNA results. Therefore, we stratified the analysis by timing of the sample at 6 weeks and 6 months.

In univariate analyses, at 6 weeks, each 10-fold increase in breastmilk cell-free or cell-associated levels per ml was associated with significantly increased hazard of postnatal HIV-1 transmission [HR 1.63 and 3.38, respectively] (Table 2). Multivariably, DNA was more important for HIV-1 transmission than RNA [aHR 2.47 vs. 1.52].

**Table 2:** Risk factors for HIV-1 transmission through breastfeeding.

	First sample taken at 6 weeks				Second sample close to ETT (or at 6 months)			
	Univariable analysis		Multivariable analysis		Univariable analysis		Multivariable analysis	
Variable	HR (95% CI)	*P*	aHR (95% CI)	*P*	HR (95% CI)	*P*	aHR (95% CI)	*P*
RNA load (for each additionallog_10_ copies/ml)	1.63 (1.33–1.98)	<0.001	1.52 (1.17–1.96)	0.002	2.65 (1.87–3.76)	<0.001	2.53 (1.64–3.92)	<0.001
DNA load (for each additionallog_10_ copies/ml)	3.38 (1.92–5.93)	<0.001	2.47 (1.33–4.59)	0.004	2.72 (1.55–4.77)	<0.001	1.73 (0.94–3.19)	0.077
Antenatal CD4 count (for each additional 100 cell/µL)	0.91 (0.79–1.04)	0.173	1.09 (0.97–1.22)	0.147	0.91 (0.79–1.04)	0.173	1.09 (0.96–1.24)	0.200
Antenatal viral load (for each additional log_10_ copies/ml)	2.11 (1.30–3.43)	0.002	1.39 (0.79–2.45)	0.252	2.11 (1.30–3.43)	0.002	1.09 (0.60–1.97)	0.777

Estimated by Cox regression with adjustment on the other factors reported in the table.

Abbreviations: HR, hazard ratio; CI, confidence interval; aHR, adjusted hazard ratio; ETT estimated time of transmission.

At 6 months, each 10-fold increase in breastmilk cell-free or cell-associated levels per ml was univariately associated with an almost 3-fold significantly increased hazard of postnatal HIV-1 transmission (Table 2). However, multivariably, the association was stronger for RNA than DNA levels [aHR 2.53 vs. 1.73].

## Discussion

We examined the prevalence of detectable RNA and DNA and levels of cell-free and cell-associated HIV-1 and associated risk of postnatal transmission at 6 weeks and 6 months in a case-control study nested in a cohort of HIV-infected mothers in KwaZulu-Natal. We showed higher median levels of cell-free than cell-associated HIV-1 virus per milliliter in breastmilk at 6 weeks and 6 months, with similar levels between breasts. Both cell-free and cell-associated virus levels in breastmilk were significantly associated with HIV-1 transmission, with a suggestion that cell-associated virus levels per milliliter may be more strongly associated with transmission than cell-free virus levels per milliliter at 6 weeks and less so at 6 months.

The prevalence of detectable HIV-1 cell-free virus in all breastmilk samples was comparable to that in previous African studies [12], [18], [31] as was that of cell-associated virus [35], [36]. Among the HIV-1 transmitting mothers, the prevalence of cell-associated virus in our study was similar to a Ugandan study at 6 weeks postpartum (77% vs. 80%, respectively) [37]. Our study showed no statistically significant difference between breasts in the levels of HIV-1 cell-free and cell-associated virus, and confirms a strong correlation of HIV-1 cell-free and cell-associated virus in breastmilk [19], [38]. This would suggest that breastmilk samples can be collected from either breast in future studies investigating HIV-1 shedding in breastmilk. Our study also confirms a positive correlation between breastmilk HIV-1 RNA and DNA and maternal antenatal plasma viral load and a negative correlation with maternal antenatal CD4 cell count [11], [12].

Previous studies have suggested that cell-associated virus levels in breastmilk decline over time [35], [36] whereas cell-free virus levels increases [39]. Overall, we show a marginally declining trend in both cell-free and cell-associated virus levels in breastmilk starting at 6 weeks postpartum [12], [36], although mothers transmitting HIV-1 had significantly higher levels of cell-free and cell-associated virus over time, as seen elsewhere [11].

Breastmilk cell-free and cell-associated levels (per ml) were significantly associated with postnatal HIV-1 transmission both univariately and multivariately. The overall adjusted model showed a 2-fold increased risk of HIV-1 transmission through breastmilk with each 10-fold increase in RNA or DNA levels as previously reported [12], [18], [19]. This is in line with results from a study in Nairobi which reported a significant association between the infected breastmilk cells and the risk of HIV-1 transmission during or after delivery [11]. Unlike our study, almost two-thirds of the first breastmilk samples in that study were collected less than 10 days after birth. Additionally, we show that at 6 weeks, DNA was more strongly associated with postnatal HIV-1 transmission than RNA while at 6 months, RNA was more strongly associated than DNA; few studies have compared RNA and DNA levels and the risk of postnatal HIV-1 transmission in the same population in early postpartum. Our results suggest that breastmilk cell-associated levels decrease earlier than noted in previous studies investigating HIV-1 transmission 9 months post-delivery [18], [40]. Future studies investigating cell-associated virus levels - especially distinguishing latently non-producing infected cells from activated producing cells - and HIV-1 transmission during lactation should be designed to focus on early life.

The current prevention of mother-to-child transmission (PMTCT) guidelines in South Africa recommend zidovudine (AZT) from 14 weeks of pregnancy, sdNVP and 3-hourly AZT intrapartum, and a single dose of tenofovir and emtracitabine postpartum, for women not eligible for lifelong ART. Their infants receive daily NVP for 6 weeks and then for up to one year during breastfeeding. Women with CD4 below 350 are eligible for lifelong ART and their infants get 6 weeks daily NVP only [41]. However, during the study period, only sdNVP was available for HIV infected women during labour/delivery and for their newborns immediately postnatally [42]. Previous studies suggest that sdNVP may reduce early postnatal HIV-1 transmission [43], as the drug has a long half-life and can be found in maternal plasma and breastmilk up to 3 weeks postpartum [44], and may reduce cell-free virus levels in the early postpartum period [45]. NVP also has a good penetration in anatomic compartments leading to reduced levels of HIV-1 plasma viral loads [46]. In our study, the estimated risk of HIV-1 transmission associated with RNA relate to samples taken at 6 weeks after perinatal sdNVP exposure, while the 6 months samples are in the absence of ART, which may partly explain the higher risk of transmission associated with RNA at 6 months. In our primary study, without ART, the HIV-1 transmission rate was 14.1% at 6 weeks and 19.5% at 6 months in exclusively breastfed infants [23]. In the recent clinical trials, where HIV-infected pregnant women took triple-ARV regimen from about 28 weeks in pregnancy (or after delivery) to 6 months postpartum, HIV transmission ranged from 3.3%–4.2% at 6 weeks and from 1.1%–8.2% at 6 months [47]–[51]. These findings suggest that giving breastfeeding women a triple-ARV regimen is safe and feasible to reduce MTCT in resource-limited settings.

However, there are suggestions that the effect of ART is different on cell-free and cell-associated virus in breastmilk [20]. Results from two separate clinical trials comparing HIV-1 cell-free and cell-associated virus in breastmilk suggest that triple-ARV regimen during pregnancy or after delivery suppressed cell-free but not cell-associated HIV-1 loads in breastmilk [33], [52]. The undetectable HIV-1 RNA in both plasma and breastmilk has been interpreted as reflecting the cessation of viral replication within maternal lymphoid tissues [53] and in the mammary gland [54]. As cell-associated HIV-1 virus in breastmilk is associated with HIV-1 transmission through breastfeeding [11], [18], their detection in breastmilk of untreated as well as those receiving antiretroviral therapy might be responsible for a residual breastmilk transmission with maternal ART.

Opportunistic infections such as congenital cytomegalovirus during pregnancy or delivery, mastitis and breast abscess have been found to be associated with the risk of HIV transmission intrapartum or postpartum [16], [55]. However, in our study, serious breast health problems were rare and there were no significant differences between HIV-infected and uninfected women [56].

The strengths of our study include the large number of breastmilk samples in the first six months postpartum and the concurrent measurement of cell-free and cell-associated virus in the right and left breasts. These findings, from a study conducted before ART was available in public health programmes in South Africa, increase understanding of the mechanisms of postnatal transmission, important for optimizing delivery of interventions in the current period.

In summary, cell-associated virus load in breastmilk is a stronger predictor of the risk of early postnatal HIV-1 infection than cell-free virus loads, independent of antenatal CD4 cell count and plasma viral loads. In contrast, cell-free virus load is a stronger predictor of later postnatal HIV-1 transmission. In contemporary breastfeeding populations with access to antiretroviral prophylaxis and ART, the residual HIV-1 transmission risk especially in the early postpartum period is partly explained by the persistence of cell-associated virus in breastmilk, and highlights a potential challenge of resource-limited settings to achieve the current UNAIDS goal for 2015 of eliminating new vertical transmission [57]. More studies are therefore needed to further knowledge on the mechanism of HIV-1 transmission during lactation and to help develop more effective drugs for use in resource-limited populations where avoidance of breastfeeding is almost impossible.
